# Effective Component Compatibility of Bufei Yishen Formula III Which Regulates the Mucus Hypersecretion of COPD Rats via the miR-146a-5p/EGFR/MEK/ERK Pathway

**DOI:** 10.1155/2022/9423435

**Published:** 2022-12-28

**Authors:** Jindi Ma, Xuefang Liu, Yumeng Wei, Ruilong Lu, Kexin Xu, Yange Tian, Jiansheng Li

**Affiliations:** ^1^Collaborative Innovation Center for Chinese Medicine and Respiratory Diseases Co-Constructed By Henan Province & Education Ministry of China, Henan University of Chinese Medicine, Zhengzhou 450046, Henan, China; ^2^Traditional Chinese Medicine (Zhong Jing) School, Henan University of Chines Medicine, Zhengzhou 450046, Henan, China; ^3^Henan Key Laboratory of Chinese Medicine for Respiratory Disease, Henan University of Chinese Medicine, Zhengzhou 450046, Henan, China

## Abstract

**Background:**

The effective-component compatibility of Bufei Yishen formula III (ECC-BYF III) with 5 ingredients (ginsenoside Rh1, astragaloside, icariin, nobiletin, and paeonol) has been shown to protect against chronic obstructive pulmonary disease (COPD). The present study aimed to observe the effects of ECC-BYF III in a COPD rat model and dissect its potential mechanisms in regulating mucus hypersecretion via the miR-146a-5p/epidermal growth factor receptor (EGFR)/MEK/ERK pathway.

**Methods:**

COPD model rats were treated with normal saline, ECC-BYF III, or N-acetylcysteine (NAC). Pulmonary function, lung tissue histology with H & E and AB-PAS staining, expression levels of interleukin (IL)-4, IL-6, IL-1*β*, MUC5AC, MUC5B, and FOXA2 in lung tissues and the mRNA and proteins involved in the miR-146a-5p/EGFR/MEK/ERK pathway were evaluated.

**Results:**

The COPD rats showed a significant decrease in the pulmonary function and serious pathological damage to the lung tissue. ECC-BYF III and NAC significantly improved the ventilation function and small airway pathological damage in the COPD rats. The goblet cells and the expression levels of IL-1*β*, IL-6, MUC5AC, and MUC5B were increased in the COPD rats and were significantly decreased after ECC-BYF III or NAC intervention. The expression levels of IL-4 and FOXA2 in the COPD rats were markedly decreased and were improved in the ECC-BYF III and NAC groups. ECC-BYF III appeared to have a potent effect in restoring the reduced expression of miR-146a-5p. The increased phosphorylation levels of EGFR, MEK, and ERK1/2 and the protein expression levels of SPDEF in the lungs of COPD rats could be significantly reduced by ECC-BYF III.

**Conclusions:**

ECC-BYF III has a significant effect in improving the airway mucus hypersecretion in COPD model rats, as well as a protective effect against limited pulmonary function and injured lung histopathology. The protective effect of ECC-BYF III in reducing airway mucus hypersecretion in COPD may involve the miR-146a-5p/EGFR/MEK/ERK pathway.

## 1. Introduction

Chronic obstructive pulmonary disease (COPD) is a progressive respiratory disease whose symptoms include dyspnoea, cough, and/or sputum production [1]. COPD affects over 250 million people globally and is now one of the top three causes of death worldwide [2, 3]. The disease causes heavy social and economic burdens, and its burden is expected to rise in the coming years. Taking China as an example, the direct medical cost of COPD ranged from 72 to 3,565 USD per capita per year, accounting for 33.33%–118.09% of the local average annual income [4]. Improving the quality of life and economic status of patients affected by the disease, delaying the progression of COPD, preventing exacerbations, and reducing the risk of comorbidities are necessary [5].

Chronic mucus hypersecretion (CMH) is one of the features of COPD. The mucus accumulation in COPD patients affects several important outcomes, such as lung function, health-related quality of life, acute exacerbations of COPD (AECOPD), hospitalizations, and mortality [6]. There is no doubt that the effective control of CMH has a beneficial effect on patients with COPD. The mechanism of CMH in COPD is complicated, but the primary mechanisms are overproduction and hypersecretion by goblet cells and the decreased elimination of mucus [7].

MUC5AC and MUC5B are principal components of airway mucus [8]. MUC5AC contributes to severe muco-obstructive lung diseases, worsening COPD pathogenesis. Various pathways are implicated in aberrant mucus production [9]. Recent studies have shown that microRNA (miR)-mRNA regulatory networks are important regulatory mechanisms for COPD CMH [10, 11]. For example, miR-31-5p is a regulator of CMH in COPD, miR-145 downregulates MUC5AC to alleviate airway remodelling and targets EGFR to inhibit the cytokine expression, and miR-330 regulates interleukin-13-induced MUC5AC secretion by targeting Munc18b in human bronchial epithelial cells [12–14]. In addition, the expression of miR-146a-5p in bronchial biopsies is inversely correlated with CMH in COPD, highlighting miR-146a-5p as a consistent regulator of mucus production in respiratory diseases [10, 15–17]. Therefore, restoring airway mucus homeostasis by regulating miR-mRNA may be an important strategy for the treatment of COPD.

Current prevention and maintenance approaches to COPD involve smoking cessation and pharmacological therapy to reduce COPD symptoms and exacerbations and improve health status and exercise endurance. The classes of medications commonly used for COPD include bronchodilators, antimuscarinic drugs, methylxanthines, anti-inflammatory agents, and inhaled corticosteroids (ICS) [2]. It is worth noting that since CMH is one of the important factors in the progression of COPD, mucolytics such as carbocysteine and N-acetylcysteine (NAC) are also used to reduce exacerbations and improve health status [18, 19].

In addition, traditional Chinese medicine (TCM), as one of the most popular complementary and alternative therapies for COPD, has shown beneficial effects. It has been used to control symptoms similar to those in COPD, for instance, cough, sputum, or shortness of breath, for thousands of years [20–22]. According to the theory of TCM, COPD belongs to the category of lung distention (Feizhang disease) [23]. A TCM formula, Bufei Yishen formula (BYF), has long been used, and beneficial effects have been shown in reducing the frequency and duration of AECOPD and improving the exercise capacity and psychosocial function of COPD patients [24, 25].

BYF is effective, but as a Chinese herbal formula, BYF encounters difficulties in standardization, modernization, and internationalization due to its complex composition. Therefore, based on network pharmacology and in vitro and in vivo experimental verifications, previous work has been performed to explore the active ingredients of BYF [24, 26]. ECC-BYF I, a combination of 10 compounds consisting of total ginsenosides, Astragalus polysaccharide, astragaloside IV, icariin, schisandrin B, nobiletin, hesperidin, peimine, paeoniflorin, and paeonol, is the core component of BYF. For the high dose (19.74 mg/kg), medium dose (9.87 mg/kg), and low dose (4.94 mg/kg) of ECC-BYF I, comprehensive evaluation of the lung function and/or inflammatory response, protease expression, and oxidative stress status results showed that there were no significant differences between high- and medium-dose ECC-BYF I and BYF [27]. Considering the safety and cost of the drug, the medium dose was chosen for the follow-up study and was further optimized to ECC-BYF II with only five components (paeonol, icariin, nobiletin, total ginsenoside, and astragaloside IV; 6.07 mg/kg). Studies have shown that ECC-BYF II has beneficial effects equivalent to those of BYF and ECC-BYF I. ECC-BYF II significantly inhibits mucus hypersecretion, which may be related to the regulation of the EGFR/PI3K/mTOR pathway [27, 28]. Since the composition of the total ginsenoside is still unclear, based on research screening, ECC-BYF II was further optimized to ECC-BYF III with 5 ingredients (ginsenoside Rh1, astragaloside, icariin, nobiletin, and paeonol; 5.5 mg/kg), and ECC-BYF III showed protective effects against COPD through anti-inflammatory and antioxidation effects [29, 30].

**Figure 1:**
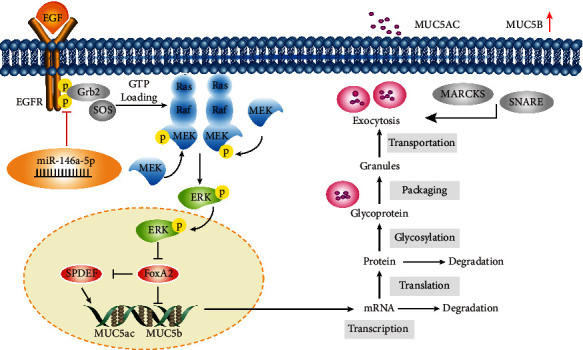
A schematic diagram of the miR-146a-5p/EGFR/MEK/ERK pathway. EGF: epidermal growth factor; Grb2: growth factor receptor-bound protein 2; FOXA2: forkhead box protein A2; MARCKS: myristoylated alanine-rich C kinase substrate; SNARE: soluble N-ethylmaleimide-sensitive fusion protein attachment protein receptor.

In this work, in view of the beneficial effects of ECC-BYF III on COPD, we explored the effects of ECC-BYF III on the CMH of COPD rats in terms of lung tissue pathology, pulmonary function, inflammatory response, goblet cells, and mucus. Moreover, since a previous study confirmed that EGFR was a direct target gene of miR-146a-5p [31], we hypothesized that the miR-146a-5p/EGFR/MEK/ERK pathway is an important mechanism for regulating CMH (Figure 1). We also initially explored the regulatory mechanism of ECC-BYF III from miR-146a-5p and its downstream proteins. These results may elucidate the effects and the possible mechanisms of E CC-BYF III on CMH in COPD rats and lay a foundation for its clinical application in COPD.

## 2. Materials and Methods

### 2.1. Preparation of Drugs

ECC-BYF III is composed of 5 ingredients, including ginsenoside Rh1 (PubChem CID: 12855920, Molecular Formula: C_36_H_62_O_9_); astragaloside IV (PubChem CID: 13943297, Molecular Formula: C_36_H_62_O_9_); icariin (PubChem CID: 5318997, Molecular Formula: C_33_H_40_O_15_); nobiletin (PubChem CID: 72344, Molecular Formula: C_21_H_22_O_8_); and paeonol (PubChem CID: 11092, Molecular Formula: C_9_H_10_O_3_). N-acetylcysteine (NAC, national medicine permission number: H20080326, 200 mg/tablet) was provided by Hainan Zambon Pharmaceutical Co. Ltd. (Hainan, China).

### 2.2. COPD Model Preparation, Administration, and Sample Collection

Sprague Dawley rats (male, 2-3 months) were purchased from the Experimental Animal Center of Shandong Province, Jinan, China. Thirty-two rats were randomized into the normal model, ECC-BYF III and N-acetylcysteine (NAC) groups. After 7 days of adapting to the environment with free access to sterile food and water, the COPD model rats (the model, ECC-BYF III and NAC groups) were exposed to cigarette smoke (Hongqiqu® filter cigarettes; Henan Tobacco Industry, Zhengzhou, China; smoke density: 3000 ± 500 ppm; 40 min twice daily) and bacteria (*Klebsiella pneumoniae* solution 0.1 mL, 6 × 10^8^ CFU/mL; every 5 days) from weeks 1 to 8, and the normal group rats were exposed to fresh air and received 0.1 mL saline solution every 5 days [28, 32]. From week 9 to week 16, the ECC-BYF III group was intragastrically administered ECC-BYF III (5.5 mg/kg/d), and the NAC group was administered N-acetylcysteine (54 mg/kg/d). The equivalent dosage of NAC was calculated according to the formula **D**_**rat**_ = **D**_**human**_ × (**I**_**rat**_**/I**_**human**_) × (**W**_**rat**_**/W**_**human**_)^2/3^ (*D*: dose; *I*: body shape index; and *W*: body weight). Simultaneously, the normal and model groups received 2 mL of 0.9% intragastric saline solution twice daily. The frequency (fR), tidal volume (*V*_*T*_), and peak expiratory flow (PEF) were detected using the unrestrained pulmonary function testing plethysmographs (Buxco Inc. USA) every fourth week.

**Figure 2:**
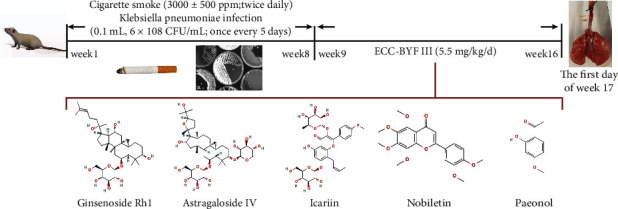
The flow chart of COPD model preparation and animal treatment.

Finally, all rats were anaesthetized on the first day of week 17 with 1% pentobarbital sodium salt (Fatal-Plus Solution, Qingdao Henley Co., Ltd., Qingdao, China; 35 mg/kg; ip), followed by pulmonary function testing and then exsanguination through the abdominal aorta. Forced vital capacity (FVC) and forced expiratory volume at 0.1 second (FEV 0.1) were measured using the FinePointe™ series PFT system (Buxco Inc. USA). The flow chart of the COPD model preparation and treatments is shown in Figure 2.

Lung tissues of the right lower lobe were sampled and cut into 3-millimetre-thick slices and then fixed in 4% paraformaldehyde for 3 days. Next, the lung tissues were embedded in paraffin for haematoxylin-eosin (H & E) processing, Alcian blue/periodic acid-Schiff (AB-PAS) staining, immunohistochemistry, immunofluorescence, and in-situ hybridization. Lung tissues of the left lobe were sampled for quantitative real-time PCR (qRT-PCR) and western blotting.

### 2.3. Lung Tissue Morphology

The lung tissue morphology was reflected by H & E staining and AB-PAS staining. The alveolar mean linear intercept (MLI, *μ*m) and the mean alveolar number (MAN/mm^2^) were counted as described in the previous literature to assess the status of emphysema in COPD rats [33]. For AB-PAS staining, the ratio of the positive staining area to its corresponding bronchial epithelial area was measured by the Image-Pro Plus 6.0 (IPP 6.0) software to evaluate the rate of goblet cells.

### 2.4. Analyses of Inflammatory Factors and Mucins

The expression levels of IL-4, IL-6, IL-1*β*, MUC5AC, MUC5B, and FOXA2 in lung tissues were detected by immunohistochemistry. A ready-to-use SABC-POD (rabbit IgG) (SA1022; Boster Bioengineering, Wuhan, China) was used for immunohistochemistry. The antibodies used for immunohistochemistry are shown in Supplementary Table 1. The integral optical density (IOD) was measured using IPP 6.0 software as previously described [34]. The location and expression of MUC5AC and MUC5B were also detected by immunofluorescence, and ImageJ software was used to analyse the fluorescence intensity.

### 2.5. Analyses of miR-146a-5p with In Situ Hybridization and qRT-PCR

In situ hybridization was used to detect the expression and location of miR-146a-5p. After dewaxing, dehydration and digestion with proteinase K (20 *µ*g/ml) working solution, 3% methanol-H_2_O_2_ was added to block endogenous peroxidase. Each section was incubated with prehybridization solution for 1 hour at 37°C, followed by the miR-146a-5p probe hybridization solution at a concentration of 500 nM overnight at 42°C. The probe of miR-146a-5p was designed as 5′-AACCCATGGAATTCAGTTCTCA-3′. After washing, the hybridization solution containing imaging oligo (DIG) (Dilution ratio: 1 : 400) was used. Then, the blocking solution (rabbit serum) was added to the section, and thirty minutes later, it was replaced with mouse antidigoxigenin-labelled peroxidase (anti-DIG-HRP). Next, the freshly prepared tyramide chromogenic reagent was added and incubated in the dark for 5 minutes at room temperature. After washing, the sections were incubated with DAPI for 8 minutes in the dark to stain the cell nuclei. Images were acquired using a Nikon fluorescence microscope and imaging system. The nuclei stained by DAPI were blue under ultraviolet excitation, and the positive expression was red.

The amount of miR-146a-5p mRNA in the lung tissues was analysed using qRT-PCR. The total RNA extraction was performed according to the conventional extraction procedures by using TRIzol™ Reagent (Code No. 15596018; Invitrogen, United States). The primers were designed and synthesized by Genewiz (Suzhou, China). The reverse transcription was conducted using the cDNA synthesis kit for realtime PCR (Code No. FSQ-101; TOYOBO, Japan). The reaction systems were performed using a fluorescence quantitative PCR System (TL-988; Xian, China). The initial predenaturation step was at 95°C for 3 min, followed by 40 cycles of 95°C for 5 s and 60°C for 34 s. At the end of the qRT-PCR, the melting curve range was set at 95°C for 15 s, 60°C for 60 s, 95°C for 15 s, and then 60°C for 15 s. The relative expression of miR-146a-5p was analysed using the 2^−ΔΔCt^ method and normalized to U6 snRNA.

### 2.6. Western Blotting

The protein expression levels of EGFR, MEK, ERK1/2, FOXA2, SPDEF, phosphorylated EGFR (p-EGFR), phosphorylated MEK (p-MEK), and phosphorylated ERK1/2 (p-ERK1/2) in the lung were tested using western blotting. After the total protein extraction according to the conventional procedure, a BCA protein assay kit (Solarbio, Beijing, China) was used to detect the protein concentrations. Denatured protein (40 *μ*g) was separated by 10% SDS-PAGE and electrotransferred to polyvinylidene difluoride (PVDF) membranes (Millipore, Bedford, USA). Membranes were blocked with 5% nonfat dry milk and incubated with the primary antibodies shown in Supplementary Table 2. After the membranes were transferred to blocking solution for 2 hours and incubated with the secondary antibody, the signals were visualized using super ECL plus reagent (Solarbio, Beijing, China).

### 2.7. Statistical Analysis

Data were analysed using IBM SPSS 22.0 software, and the results are expressed as the mean ± standard deviation. One-way analysis of variance (ANOVA) was employed for multiple comparisons. The significance level was set as *P* < 0.05.

## 3. Results

### 3.1. ECC-BYF III Improved Pulmonary Function and Pathological Injury in COPD Rats

To confirm that the COPD model was successfully established by the exposure to cigarettes and *Klebsiella pneumonia* and to confirm the therapeutic effects of ECC-BYF III, unrestrained pulmonary function was detected by whole-body plethysmography every fourth week. The frequency of breathing of the rats in the control group generally showed a downwards trend with increasing age, while the frequency of the rats in the model group increased compared with the control. In the ECC-BYF III group, the frequency was lower than that in the model group, but there was no significant difference. With increasing age, the VT and PEF of the rats in the control group increased. After the completion of COPD model preparation in the 8^th^ week, the VT and PEF of the COPD rats decreased significantly (*P* < 0.05). At the 16^th^ week, after eight weeks of the ECC-BYF III intervention, the VT and PEF in the ECC-BYF III group were significantly increased compared to those in the COPD group (*P* < 0.05).

**Figure 3:**
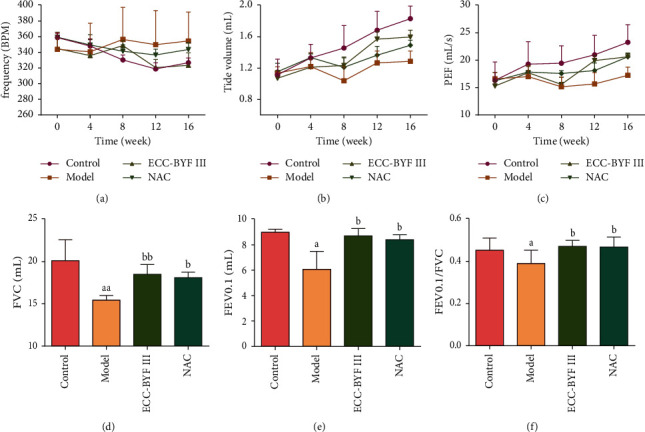
ECC-BYF III improved the pulmonary function in COPD rats. (a–c) Changes in the frequency (fR), tidal volume (VT), and peak expiratory flow (PEF) in all groups (*n* = 8). (d–f) Changes in invasive pulmonary function (FVC, FEV0.1 and FEV0.1/FVC) in all groups (*n* = 6). The control refers to healthy control rats; the model refers to COPD rats; ECC-BYF III refers to ECC-BYF III—treated COPD rats and NAC refers to N-acetylcysteine-treated COPD rats. All data are presented as the mean ± (S) ^a^*P* < 0.05 versus the control group, ^aa^*P* < 0.01 versus the control group, ^b^*P* < 0.05 versus the model group, and ^bb^*P* < 0.01 versus the model group.

Invasive pulmonary function by utilizing an invasive test system (PFT) was assessed before sampling. The FVC, FEV0.1, and FEV0.1/FVC in the treatment groups (ECC-BYF III and NAC) showed significant improvement compared with those in the model (*P* < 0.05, *P* < 0.01) (Figures 3(a)–3(f)). These data indicate airflow limitations in the COPD rats, and ECC-BYF III effectively improved the ventilation function of the model rats.

**Figure 4:**
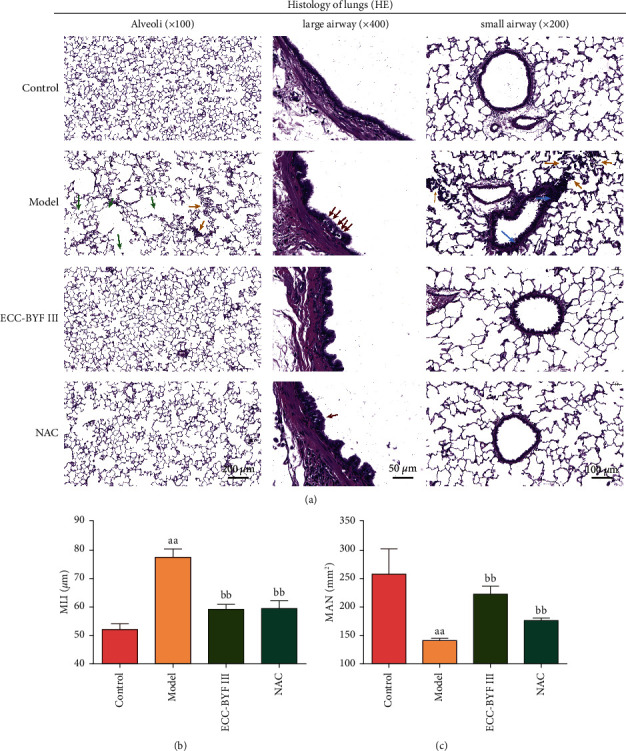
ECC-BYF III improved the pathological changes in COPD rats. (a) The representative image of the lung tissue pathology (HE; alveoli, 100×, scale bar: 200 *μ*m; large airway, 400×, scale bar: 50 *μ*m; small airway, 200×, scale bar: 100 *μ*m). The green arrows indicate the fracture and fusion of the alveolar wall, orange arrows indicate inflammatory cell infiltration, red arrows indicate goblet cells, and blue arrows indicate small airway wall thickening. (b, c) Changes in the mean linear intercept (MLI) and mean alveolar number (MAN). All data are presented as the mean ± *S* (*n* = 6). ^aa^*P* < 0.01 versus the control group and ^bb^*P* < 0.01 versus the model group.

For pathological changes, the characterization of COPD has not only the traditional distinctions of “emphysema” and “chronic bronchitis” based on the presence and type of emphysema but also small airway disease, bronchial wall dilatation, wall thickening, and large airway disease [35, 36]. We mainly observed changes in the alveoli, large airways, and small airways. The size of the alveoli in the rats of the control group was relatively uniform, and the structure of the alveoli was complete. In addition, the inflammatory cell infiltration was rarely observed. In the rats of the model group, many of the alveolar walls were broken, and some alveoli could be seen fused into bullae (Figure 4(a)). MAN and MLI showed that the diameter of the alveoli in the model group was significantly increased compared with that in the control group, and the number of alveoli per unit area was significantly reduced (*P* < 0.01) (Figures 4(b) and 4(c)).

Furthermore, a large amount of inflammatory cell infiltration was observed in the lung parenchyma. The large airways and small airways of the rats in the model group also showed significant changes. More goblet cells were observed in the airway epithelial cells, and more inflammatory cells were observed around the small airway, with wall dilatation and thickening. The rats of the ECC-BYF III group showed an improvement with a complete alveolar structure, a more uniform size of alveoli, less thickening of the bronchiolar wall, as well as fewer inflammatory cells, and fewer goblet cells compared with those in the model group. In summary, the pathological changes of the model showed obvious inflammatory cell infiltration, with the alveolar cavity enlargement signs of moderate and severe emphysema, and ECC-BYF III significantly improved the abovementioned pathological damage to the lung tissue.

### 3.2. ECC-BYF III Alleviated the Local Inflammatory Response and Airway Mucus Hypersecretion in the Lung Tissue of COPD Rats

**Figure 5:**
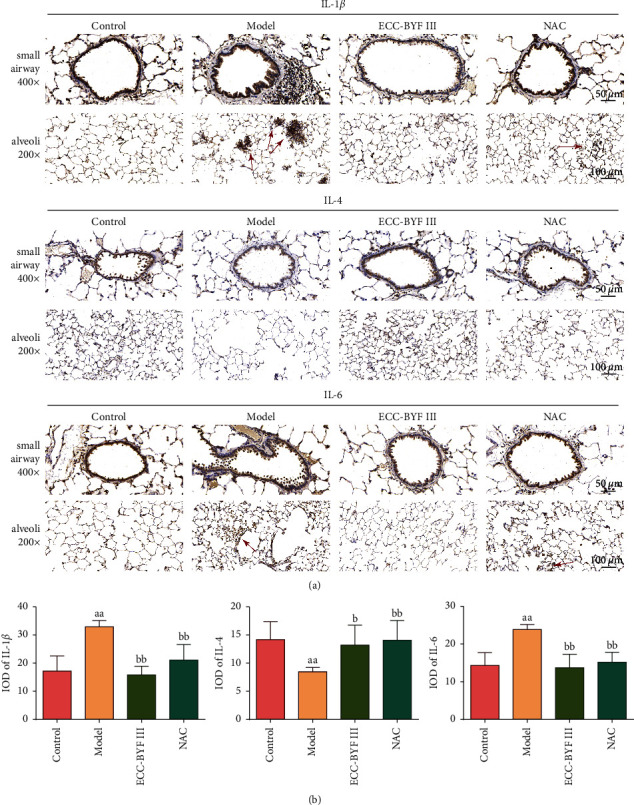
ECC-BYF III reduced the lung inflammation in COPD rats. (a) The photograph of the changes in IL-1*β*, IL-4, and IL-6 in the lung tissue (IHC; 200×, 400×). The red arrows indicate the inflammatory cell infiltration. (b) Changes in the IOD of IL-1*β*, IL-4, and IL-6 in all groups. All data are presented as the mean ± *S* (*n* = 6). ^aa^*P* < 0.01 versus the control group, ^b^*P* < 0.05 versus the model group, and ^bb^*P* < 0.01 versus the model group.

COPD is a chronic inflammatory disease in which inflammatory cells infiltrate the bronchial mucosa and lung parenchyma [37, 38]. It is worth mentioning that the airway also responds to the inflammation with increased mucus secretion [39]. Therefore, the localization and expression of inflammatory factors in the lung tissue were observed. The expressions of IL-1*β* and IL-6 in COPD rat lung tissues were significantly increased. A large number of positively stained inflammatory cytokines could be seen in the lung parenchyma and around the small airways. After the administration of ECC-BYF III or NAC, inflammatory cytokine infiltration was markedly decreased (*P* < 0.01). At the same time, the expression of IL-4, which has an inhibitory effect on inflammation, was reduced in the lung tissue of model rats and recovered after the treatment (*P* < 0.05) (Figures 5(a) and 5(b)).

**Figure 6:**
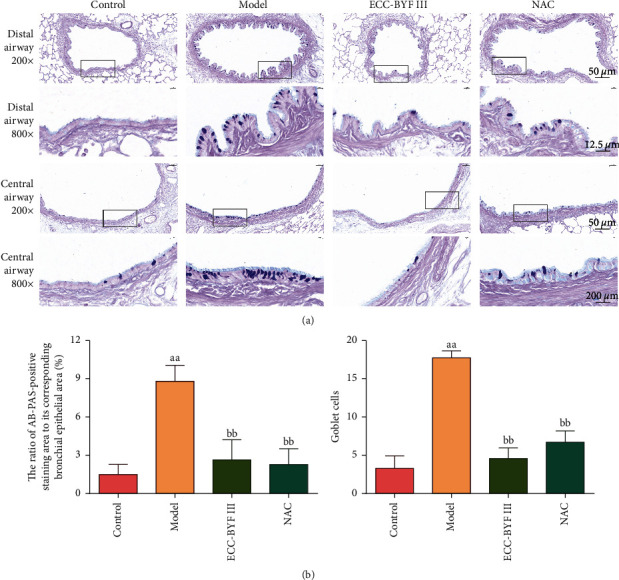
ECC-BYF III reduced the goblet cell density in COPD rats. (a) The photograph of the changes in the goblet cells in the airway (AB-PAS; 200×, 800×); (b) the ratio of the AB-PAS-positive staining area to its corresponding bronchial epithelial area (%); the number of goblet cells per unit field of view. Data are presented as the mean ± *S* (*n* = 6). ^aa^*P* < 0.01 versus the control group and ^bb^*P* < 0.01 versus the model group.

Since the hypersecretion of airway mucus is an important pathological factor in COPD, this study focused on the effects and potential mechanisms of ECC-BYF III on COPD by alleviating CMH. Goblet cells are essential to secrete mucus, and the goblet cell density increases in COPD patients [40]. As shown in Figure 6, the goblet cells were stained blue by AB-PAS. In the airway epithelium of the control group rats, there were a few goblet cells in the central airway and almost no goblet cells in the distal airways. Meanwhile, many blue-stained goblet cells were observed in the COPD rats, not only in the central airway but also in the distal airways (Figure 6(a)). By counting the number of goblet cells and the area of positive staining, both ECC-BYF III and NAC were found to significantly reduce the goblet cell increase caused by cigarette smoke exposure combined with the bacterial infection (*P* < 0.01) (Figure 6(b)).

**Figure 7:**
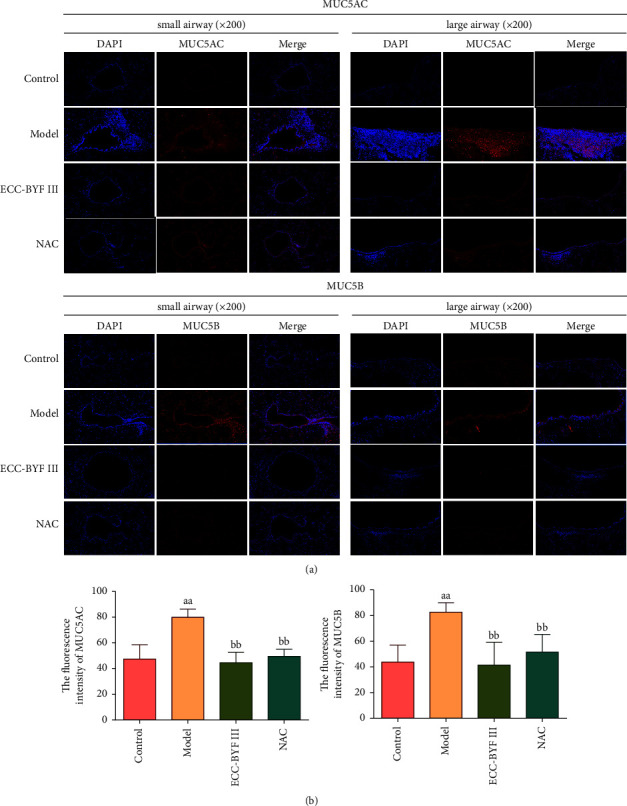
The immunofluorescence intensity of the MUC5AC and MUC5B in lung tissues. (a) The lung tissue immunofluorescence photograph of the expressions of MUC5AC and MUC5B (immunofluorescence; 200×); (b) the fluorescence intensities of MUC5AC and MUC5B. Data are presented as the mean ± *S* (*n* = 6). ^aa^*P* < 0.01 versus the control group and ^bb^*P* < 0.01 versus the model group.

**Figure 8:**
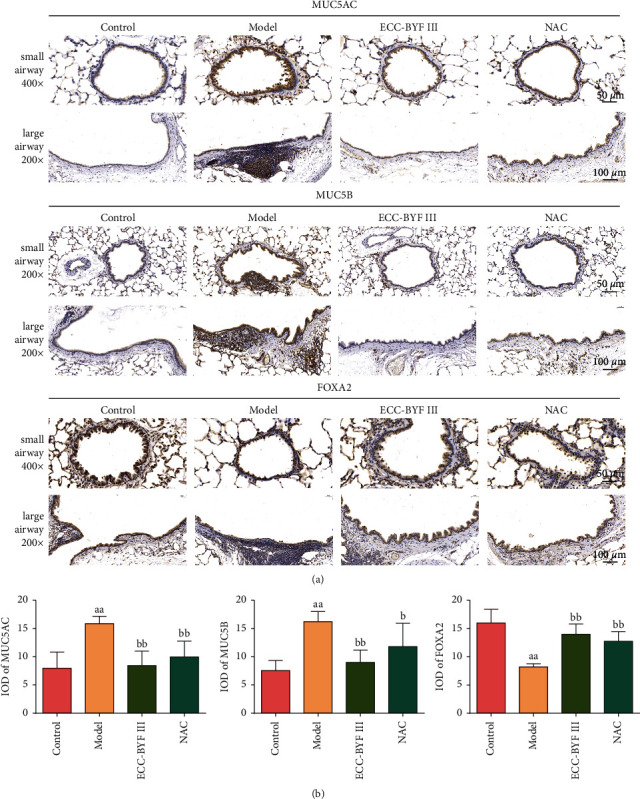
Expressions of MUC5AC, MUC5B, and FOXA2 in lung tissues. (a) The lung tissue immunohistochemistry photograph of the expressions of MUC5AC, MUC5B, and FOXA2 (immunohistochemistry; 200×, 400×); (b) integral optical density (IOD) of MUC5AC, MUC5B, and FOXA2. Data are presented as the mean ± *S* (*n* = 6). ^aa^*P* < 0.01 versus the control group, ^b^*P* < 0.05 versus the model group, and ^bb^*P* < 0.01 versus the model group.

The polymeric mucins MUC5AC and MUC5B are integral components of the airway mucus [8]. Immunohistochemistry and immunofluorescence techniques were used to detect the expression and localization of MUC5AC and MUC5B. MUC5AC is localized to goblet cells in the surface epithelium and in the terminal secretory ducts of submucosal glands, and the MUC5B protein is localized to mucous cells in submucosal glands and secretory cells within the surface airway epithelium of the trachea and bronchi [8]. The protein expressions of MUC5AC and MUC5B were low in normal rat airways. It increased markedly in the COPD rats and was obviously downregulated by ECC-BYF III and NAC (Figures 7(a), 7(b), 8(a) and 8(b)). Furthermore, one of the forkhead box family members, forkhead box protein A2 (FOXA2), participates in airway polymeric mucin expression and mucous metaplasia. It is a potent inhibitor of the goblet cell differentiation that inhibits the MUC5AC expression. In COPD rats, the expression of FOXA2 in the lung was markedly decreased, while ECC-BYF III and NAC improved its expression (*P* < 0.01) (Figures 8(a) and 8(b)). It can be seen from these data that ECC-BYF III is good at suppressing airway mucus hypersecretion in COPD rats, and its effect of reducing CHM shows no difference from NAC.

### 3.3. ECC-BYF III Regulated the miR-146a-5p/EGFR/MEK/ERK Pathway in COPD Rats

EGFR is the key to the EGFR signalling network, stimulating CMH by multiple downstream pathways [41]. In addition, recent studies have shown that the microRNA-mRNA regulatory network is related to the CMH in COPD [10]. Among several microRNAs involved in the regulation of mucus secretion, miR-146a-5p has been shown to be significantly associated with COPD [42], and more importantly, EGFR has been identified as a direct target gene of the miR-146a-5p [31]. Therefore, we initially observed changes in the miR-146a-5p in COPD rats and then detected activated proteins related to the miR-146a-5p/EGFR/MEK/ERK pathway.

**Figure 9:**
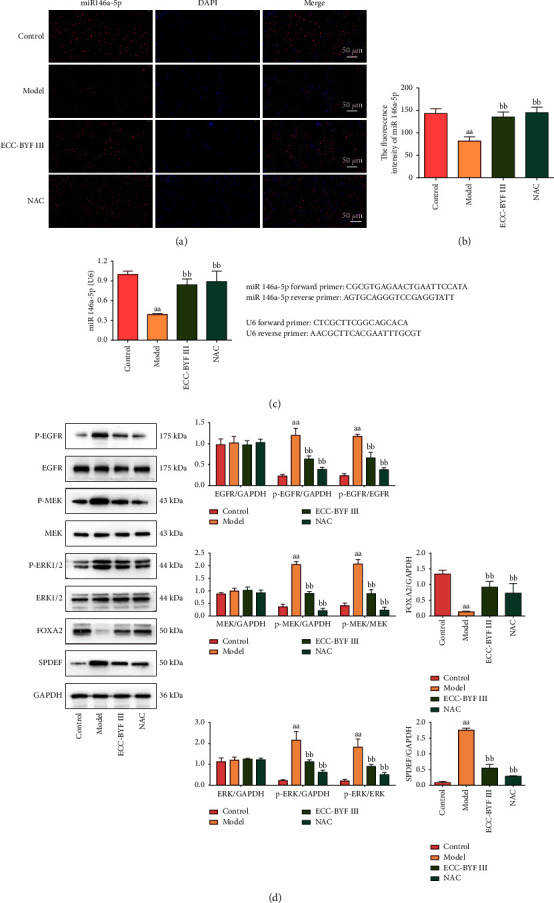
ECC-BYF III regulated the miR-146a-5p/EGFR/MEK/ERK pathway in COPD rats. (a) The immunofluorescence photograph of the expression of miR-146a-5p in the lung tissue (immunofluorescence; 200×). (b) The fluorescence intensity of miR-146a-5p. (c) The mRNA expression level of miR-146a-5p in the lung tissue of COPD rats. Data are presented as the mean ± *S* (*n* = 6). ^aa^*P* < 0.01 versus the control group and ^bb^*P* < 0.01 versus the model group. (d) The protein expression levels of p-EGFR, EGFR, p-MEK, MEK, p-ERK1/2, ERK1/2, FOXA2, and SPDEF. Data are presented as the mean ± *S* (*n* = 3). ^aa^*P* < 0.01 versus the control group and ^bb^*P* < 0.01 versus the model group.

The miR-146a-5p in-situ hybridization showed that the expression of miR-146a-5p was significantly reduced in the model rats compared with the normal rats (*P* < 0.01), while ECC-BYF III and NAC significantly increased the expression of miR-146a-5p (*P* < 0.01). The expression of miR-146a-5p mRNA detected by qRT-PCR was consistent with the results of the in-situ hybridization (Figures 9(a)–9(c)). As shown in Figure 9(d), compared with the control group rats, the phosphorylation levels of EGFR, MEK, and ERK1/2 and the protein expression levels of SPDEF in the lungs of COPD rats were all increased, and the overexpression of these proteins was significantly reduced by ECC-BYF III and NAC (*P* < 0.01). The FOXA2 expression was significantly decreased in the model group and significantly increased after ECC-BYF III or NAC intervention (*P* < 0.01).

## 4. Discussion

COPD is a major global health problem that affects approximately 384 million people worldwide, and with the ageing of the population, the prevalence is still increasing [43, 44]. With clinical symptoms such as chronic and progressive dyspnoea, chronic cough, sputum production, chest tightness and fatigue, the work and everyday life of COPD patients are seriously affected, placing a significant burden on patients and society. More seriously, COPD kills more than 3 million people worldwide every year, and few advances have been made to ameliorate disease progression or reduce mortality [45]. Therefore, more attention should be placed on controlling the aggravation and progression of COPD.

In China, a country with 100 million COPD patients, traditional Chinese medicine is widely used in the supplementary and alternative treatment of COPD, and its beneficial effects have been confirmed [20]. According to the TCM theory, one of the common pathogeneses of COPD is Qi deficiency of the lung and kidney. Therefore, the Bufei Yishen formula (BYF), with the functions of invigorating the lung and kidney, resolving phlegm, and activating blood circulation, has a good effect for COPD patients [25]. Through multiple in-vivo and in-vitro experiments, the 5 core active ingredients (ginsenoside Rh1, paeonol, icariin, nobiletin, and astragaloside IV) in BYF have been identified in previous research. The 5-ingredient formula derived from BYF was named ECC-BYF III. Although it is a medicine composed of five components, it still follows the principles of TCM syndrome differentiation and TCM compatibility, and it has equivalent therapeutic effects and safety to BYF [29].

In this study, we observed the effect of ECC-BYF III on COPD rats, which showed that ECC-BYF III could delay the decline in the lung function, reduce lung inflammation, and improve emphysema-like changes in lung tissue pathology in COPD rats. ECC-BYF III clearly has beneficial effects. We are also interested in its mechanism in COPD.

Among its complex pathological mechanisms, small airway disease is a cardinal feature of COPD [46]. Since chronic mucus hypersecretion (CMH) is one of the key characteristics and pathogenesis of COPD, we focused on it. For CMH, goblet cells deserve particular attention because uncontrolled production of goblet cells, being a hallmark feature of COPD, leads to increased production and secretion of mucins within the airways [47].

Mucus is an innate barrier against toxic chemicals and pathogens by entrapment and mucociliary clearance. However, patients with mucus obstructive lung disease experience progressive spirals of inflammation, mucostasis, airway infection, and lung function decline. A clinical study showed an increased number of mucus-occluded small airways in COPD patients, which corresponded with their disease severity [46]. MUC5AC and MUC5B are the predominant gel-forming mucins of airways, and their concentrations increase in COPD exacerbations [48, 49]. Among them, MUC5AC and COPD are more closely related. Studies have confirmed that increased MUC5AC concentrations in the airways might contribute to COPD initiation, progression, exacerbation risk, and overall pathogenesis [50]. On the other hand, inhibition of MUC5AC may ameliorate COPD exacerbations [49].

Traditional Chinese medicine not only has a good effect in improving COPD symptoms but also in reducing CMH. For instance, Guifu Dihuang pills ameliorated CMH by suppressing MUC5AC expression in COPD mice [51]. Louqin Zhisou decoction inhibits CMH in AECOPD rats by suppressing the EGFR/PI3K/AKT signalling pathway and restoring the Th17/Treg balance [52]. It is important that BYF has an expectorant effect [25, 27]. Our results showed significantly increased expression levels of MUC5AC and MUC5B and massive goblet cell metaplasia in the airways of COPD rats, while ECC-BYF III had an obvious inhibitory effect on these changes. Notably, there was no significant difference in the regulation of CMH between ECC-BYF III and NAC, a well-known and safe mucolytic agent. Considering the close relationship between mucus hypersecretion and inflammation, we also observed representative inflammatory indices IL-1*β*, IL-4, and IL-6 in the lung tissue. These results show that ECC-BYF III has a good alleviating effect on chronic lung inflammation in COPD rats.

Many pathways and molecules are involved in the regulation of airway mucus homeostasis. Recent studies have shown that microRNAs (miRNAs), a class of noncoding, short single-stranded RNAs, regulate gene expression by a post-transcriptional mechanism and have important roles in the airway mucus regulation [11]. In particular, some miRNAs show prominent roles in regulating airway mucus homeostasis. For example, it was found that miRNA-34b/c regulated mucus secretion in RSV-infected airway epithelial cells and that miRNA-330 regulated IL-13-induced MUC5AC secretion in human bronchial epithelial cells [14, 53]. In addition, tumour necrosis factor-*α* promoted airway mucus hypersecretion by inhibiting miR-146a-5p and miR-134-5p levels [54].

In the miRNA-mRNA regulatory networks underlying CMH in COPD, miR-146a-5p was also screened as a potential key link [10]. According to previous reports, repressing miR-146a-5p levels in human airway epithelial cells is associated with airway mucus hypersecretion [55]. MiR-146a-5p also plays an essential role in the aberrant epithelial-fibroblastcross-talk in COPD [16]. Furthermore, researchers have identified EGFR as a direct target gene of miR-146a-5p [31].

It is not disputed that EGFR is a key link in CMH. Studies have demonstrated that therapeutic inhibition of mucin production with an EGFR antagonist ameliorates immunopathology in an AECOPD mouse model [49]. EGFR regulates airway mucus secretion through multiple downstream pathways, among which EGFR-activated extracellular signal-regulated kinase (ERK) signalling plays a critical role in the MUC5AC induction. A study showed that the activation of the EGFR-ERK pathway contributed to sustained mucin production in COPD [56].

Therefore, we focused on miR-146a-5p and its direct target gene EGFR to explore the mechanisms by which ECC-BYF III improves mucus secretion. With the same trend as the results reported in the literature, the expression of miR-146a-5p in the lung tissue of COPD model rats decreased, and the phosphorylation levels of EGFR, MEK, and ERK1/2 increased. Regarding the above changes, ECC-BYF III effectively upregulated the expression of miR-146a-5p and inhibited the activation of the EGFR/MEK/ERK pathway. The results of this study suggest that the miR-146a-5p/EGFR/MEK/ERK pathway may be one of the action pathways of ECC-BYF III on CMH. However, there may be other mechanisms by which ECC-BYF III affects the secretion of airway mucus, and we will further explore the mechanisms of ECC-BYF III in follow-up studies to clarify them.

However, this study has a limitation in addressing the dosage and pharmacological aspects of the treatment due to the lack of high-dose and low-dose ECC-BYF III groups. Moreover, the study of the effect of ECC-BYF III on the miR-146a-5p/EGFR/MEK/ERK pathway is only preliminary. We will further explore the dose-effect relationship of ECC-BYF III on COPD to clarify the mechanisms of ECC-BYF III in the future.

## 5. Conclusions

ECC-BYF III has a good efficacy in reducing airway mucus hypersecretion and inflammation in rats with COPD induced by cigarette smoke exposure combined with bacterial infection. The mechanism may be related to the miR-146a-5p/EGFR/MEK/ERK pathway.

## Data Availability

Since ECC-BYF III is still under further study, datasets of the current study are available from the corresponding author or first author on reasonable request.

## Abbreviations

COPD: Chronic obstructive pulmonary disease
TCM: Traditional Chinese medicine
BYF: Bufei Yishen formula
ECC-BYF III: Effective-component compatibility of Bufei Yishen formula III
NAC: N-Acetylcysteine
EGFR: Epidermal growth factor receptor
CMH: Chronic mucus hypersecretion
AECOPD: Acute exacerbations of COPD
fR: The frequency
VT: Tidal volume
PEF: Peak expiratory flow
ICS: Inhaled corticosteroids
FVC: Forced vital capacity
FEV 0.1: Forced expiratory volume at 0.1 s
H & E: Haematoxylin-eosin
AB-PAS: Alcian blue/periodic acid-Schiff
MLI: Alveolar mean linear intercept
MAN: Mean alveolar number
IOD: Integral optical density.

## Supplementary Materials

Supplementary Materials Supplementary Table 1: Antibodies used in immunohistochemistry. Supplementary Table 2: Primary antibodies used in western blotting.
