# Supplementary material for: Effective Component Compatibility of Bufei Yishen Formula III Which Regulates the Mucus Hypersecretion of COPD Rats via the miR-146a-5p/EGFR/MEK/ERK Pathway
Source: Evid Based Complement Alternat Med. 2022 Dec 28;2022:9423435. doi: 10.1155/2022/9423435 (PMC9812609; doi:10.1155/2022/9423435)
Supplement: Supplementary Materials — Supplementary Table 1: Antibodies used in immunohistochemistry. Supplementary Table 2: Primary antibodies used in western blotting. [file 9423435.f1.docx]

**Supplementary Table 1 Antibodies used in immunohistochemistry**

| **Antibodies** | **Dilution ratio** | **Item number** | **Manufacturer** |
| --- | --- | --- | --- |
| Anti-IL-1β Polyclonal Antibody | 1:100 | K108840P | Solarbio |
| Anti-IL-4 Polyclonal Antibody | 1:100 | K004107P | Solarbio |
| Anti-IL-6 Polyclonal Antibody | 1:200 | K009385P | Solarbio |
| MUC5AC Polyclonal Antibody | 1:100 | E-AB-40037 | Elabscience |
| MUC5B Polyclonal Antibody | 1:200 | E-AB-15988 | Elabscience |
| Anti-FOXA2 Antibody | 1:100 | A01032-2 | Boster Bio-engineering |

**Supplementary Table 2 Primary antibodies used in western blotting**

| **Antibodies** | **Dilution ratio** | **Item number** | **Manufacturer** |
| --- | --- | --- | --- |
| Rabbit Anti-Phospho-EGFR Antibody | 1:1000 | 3777 | Cell Signaling |
| Rabbit Anti-EGFR Antibody | 1:1000 | ab52894 | Abcam |
| Rabbit Anti-Phospho-MEK1/2 Antibody | 1:1000 | 9154 | Cell Signaling |
| Rabbit Anti- MEK Antibody | 1:2000 | GTX111338 | GeneTex |
| Rabbit Anti-Phospho-ERK1/2 Antibody | 1:1000 | 4370 | CST |
| Rabbit Anti- ERK1/2 Antibody | 1:1000 | 4695 | CST |
| Rabbit Anti- FOXA2 Antibody | 1:2000 | 8186 | Cell Signaling |
| Rabbit Anti- SPDEF Antibody | 1:500 | A14114 | ABclonal |
| GAPDH Monoclonal Antibody | 1:10000 | YM3029 | Immunoway |
